# Antiosteoarthritic Effects of ChondroT in a Rat Model of Monosodium Iodoacetate-Induced Osteoarthritis

**DOI:** 10.1155/2018/8565132

**Published:** 2018-02-08

**Authors:** Kil-Joon Bae, Ji-Won Jeong, Chan-Hun Choi, Jeong-Yoon Won, Tae-Gwang Kim, Young-Ran Kim, Chang-Su Na, Seon-Jong Kim

**Affiliations:** ^1^Department of Korean Medical Rehabilitation, Mokpo Korean Medicine Hospital of Dongshin University, 313 Baengnyeon-daero, Mokpo-si 58665, Republic of Korea; ^2^College of Korean Medicine, Dongshin University, 185 Geonjae-ro, Naju-si, Jeollanam-do 58245, Republic of Korea; ^3^Department of Korean Medical Rehabilitation, Dongshin Korean Medicine Hospital, 351 Omok-ro, Yangcheon-gu, Seoul 07999, Republic of Korea; ^4^Department of Korean Medical Rehabilitation, Gwangju Korean Medicine Hospital of Dongshin University, 141, Wolsan-ro, Nam-gu, Gwangju 61619, Republic of Korea; ^5^College of Pharmacy, Chonnam National University, 77 Yongbong-ro, Buk-gu, Gwangju 61186, Republic of Korea

## Abstract

*Ganghwaljetongyeum* is a traditional Korean herbal medicine used to treat joint pain, limited motion, fever, and swelling; it also inhibits inflammatory processes associated with arthritis. ChondroT, a water extract of* Ganghwaljetongyeum*, is a new complex herbal medicine. This study investigated the effects of ChondroT using a rat model of monosodium iodoacetate- (MIA-) induced osteoarthritis. Thirty-six rats were randomly divided into three ChondroT groups and a normal, control, and positive control group. Changes in paw edema volume, histopathology, and plantar withdrawal response were analyzed. Further, inflammatory cytokines, arachidonic acids, liver and kidney function, and hematological features were measured. ChondroT significantly decreased paw edema by the 5th day and notably improved articular cartilage damage; it also significantly improved the plantar withdrawal response in terms of both reaction time and force intensity. Moreover, treatment with ChondroT significantly decreased the serum levels of tumor necrosis factor alpha, interleukin-1*β*, interleukin-6, and prostaglandin E2 and significantly increased serum aspartate aminotransferase and alanine aminotransferase levels. This study demonstrates that ChondroT has anti-inflammatory and analgesic effects in a MIA-induced osteoarthritis rat model. These results support the clinical relevance of ChondroT for future use in patients with osteoarthritis. However, further studies are required to elucidate the corresponding mechanisms.

## 1. Introduction

Osteoarthritis (OA) is a joint disease also known as degenerative bone disease, degenerative arthritis, and osteoarthrosis. It has recently been determined that OA results from various interactions after a joint has been damaged and can be classified as idiopathic or secondary; idiopathic factors include age, gender, obesity, and genetic factors, with the most powerful risk factor being age [1, 2].

GHJTY* (Ganghwaljetongyeum)*, a traditional Korean herbal medicine used to treat joint pain, limited motion, fever, and swelling, has been shown to inhibit inflammatory processes associated with arthritis [3]. Previously, we showed that GHJTY effectively attenuates rheumatoid arthritis by inhibiting the production of proinflammatory mediators and synoviocyte proliferation [4].

GHJTY is a complex herbal decoction composed of 18 plants; therefore, we previously used bioinformatics and screening experiments to select the five medicinal herbs present in GHJTY with the greatest potential to improve the efficacy and convenience of pharmaceutically prescribing GHJTY [5]. These five medicinal herbs include* Ostericum koreanum *Maximowicz (Osterici Radix; OK),* Lonicera japonica *Thunberg (Lonicerae Folium; LJ),* Clematis mandshurica *Ruprecht (Clematis Radix; CM),* Angelica gigas *Nakai (Angelicae Gigantis Radix; AG), and* Phellodendron amurense *Ruprecht (Phellodendri Cortex; PA). The five herbs were combined in a 6 : 4 : 4 : 4 : 3 ratio, respectively, and a water extract solution of these 5 herbs was termed ChondroT, a new complex herbal medicine.

ChondroT exerts chondroprotective effects and demonstrates multitarget mechanisms related to inflammation and arthritis. In addition, the suppressive effect of ChondroT was greater than that exhibited by GHJTY, suggesting that ChondroT has therapeutic potential for the treatment of arthritis [6]. In addition, ChondroT has been shown to be effective in treating a rat model of inflammatory arthritis. Specifically, ChondroT significantly suppressed the progression of complete Freund's adjuvant- (CFA-) induced arthritis based on decreased paw and knee joint swelling and was effective in preventing articular cartilage and synovial tissue degeneration. The protective mechanisms of ChondroT treatment are partially explained by a decrease in the proinflammatory cytokines tumor necrosis factor- (TNF-) *α*, interleukin- (IL-) 1*β*, and IL-6 [7].

To further investigate the efficacy, mechanism of action, and toxicity of ChondroT as an antiarthritic herbal drug, we investigated its effects in a monosodium iodoacetate- (MIA-) induced OA rat model. Inflammatory cytokines (TNF-*α*, IL-1*β*, IL-6, and prostaglandin (PG) E2) were analyzed; the knee joint articular structures were stained with hematoxylin and eosin (H&E) and safranin O-fast green; the withdrawal response of mechanical allodynia of osteoarthritic rats was observed; and the serum aminotransferase, albumin, blood urea nitrogen (BUN), and creatinine levels were measured.

## 2. Materials and Methods

### 2.1. Animals

Adult male Sprague-Dawley rats weighing 170–180 g were housed in a room with constant temperature (24–26°C) and humidity (40–60%). Food (Pellet, GMO, Korea) and water were available ad libitum. Animals were acclimated to the laboratory environment for 1 week prior to experimentation, and all procedures were approved by the Institutional Animal Care and Use Committee of the Dongshin University (DSU-2014-059).

### 2.2. MIA-Induced Arthritis and Drug Administration

Arthritis was induced by injecting 3 mg/kg MIA (Sigma, USA) into the left knee joint space of rats using a sterile hypodermic syringe (Korea Vaccine Co., Korea). MIA was dissolved in 0.9% sterile saline and administered in a volume of 50 *μ*l at the start of the experiment and 25 *μ*l after initial MIA-induction to induce swelling. Animals were then divided into 6 groups (*n* = 6/group), including a normal; MIA arthritis (control); MIA arthritis treated with 20 mg/kg of Joins Tab extract per day (Joins Tab); and MIA arthritis treated with 50, 100, or 200 mg/kg ChondroT extract per day (ChondroT 50, 100, and 200). Oral administration of ChondroT was initiated on the 8th day after arthritis induction and continued for 10 days thereafter. Animals were anesthetized using 2.5% isoflurane and hind paw and knee joint volumes were measured using a digital plethysmometer (LE7500, Panlab, Spain) 1, 3, 5, 7, 9, and 11 days after oral ChondroT administration.

### 2.3. Preparation of Herbal Materials

The five herbal medicines comprising ChondroT were purchased from Omniherb Co. (Yeongcheon, Korea), and their origin was taxonomically confirmed by Professor Jong-Kil Jeong in the Department of Herbology at the College of Korean Medicine, Dongshin University. The plant names have been checked with the plant list.

The five herbs (OK, LJ, AG, CM, and PA) were combined in a 6 : 4 : 4 : 4 : 3 ratio (Table 1). ChondroT was prepared via a single water extraction using 10-fold water solvent at 100°C for 3 h and then filtered (180 mesh). The water extract solution of ChondroT was concentrated using a continuous vacuum evaporator (approximately 55–60°C, 670 mmHg) followed by vacuum drying (720 mmHg) for 8 h. The yield was approximately 29.4%.

### 2.4. Reagents and High-Performance Liquid Chromatography (HPLC) Analysis

**Figure 1:**
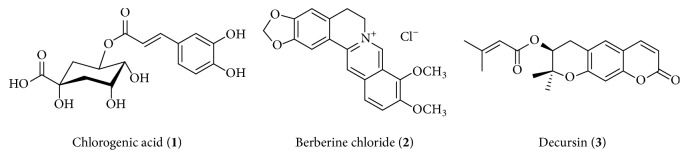
Chemical structure of compounds** 1**–**3**.

Chlorogenic acid (**1**) and berberine chloride (**2**) were purchased from the U.S. Pharmacopeial Convention (Rockville, MD, USA). Decursin (**3**) was purchased from ChemFaces Biochemical Co. Ltd. (Wuhan, China). The purity of reference compounds** 1**–**3** was 97.3, 81.0, and 99.4%, respectively, as determined by HPLC analysis; the chemical structures of compounds** 1**–**3** are shown in Figure 1. HPLC-grade methanol, acetonitrile, and water were obtained from J. T. Baker (Phillipsburg, NJ, USA). Analytical grade formic acid was purchased from Sigma-Aldrich (St. Louis, MO, USA).

For ChondroT quality control, all experiments were performed using a Shimadzu Prominence LC-20A Series (Shimadzu, Kyoto, Japan) equipped with a solvent delivery unit (LC-20AT), online degasser (DGU-20A3R), column oven (CTO-20AC), autosampler (SIL-20AC), and UV-VIS detector (SPD-20A). Data acquisition and processing were conducted using Lab Solution software (version 5.73 SP3, Kyoto, Japan). Compounds** 1**–**3** were separated using a Waters SunFire C18 column (4.6 × 250 mm; 5 *μ*m, Milford, MA, USA) maintained at 40°C. The mobile phases consisted of 0.1% (v/v) aqueous formic acid (A) and 0.1% (v/v) formic acid in acetonitrile (B). The gradation conditions were optimized as follows: a range of 0–10 min, 10–35% B; 10–30 min, 35–65% B; 30–35 min, 65–80% B; 35–40 min, 80% B; 40-41 min, 80–10% B; and 41–50 min, 10% B. Flow rate and injection volume were 1.0 mL/min and 10 *μ*L, respectively. For HPLC analysis, lyophilized ChondroT (1 g) was dissolved in 100 mL 70% methanol and extracted for 60 min by sonication. The ChondroT extract solution was centrifuged at 3,000 rpm for 10 minutes and then passed through a 0.45 *μ*m syringe filter before HPLC analysis.

### 2.5. Blood and Serum Tests

Blood samples were collected, and serum was separated from whole blood using a high-speed centrifuge (VS-600CFi, Korea) at 3500 rpm (*g* = 27.391) for 20 min; aspartate aminotransferase (AST) and alanine transaminase (ALT) levels were measured.

### 2.6. Measurement of TNF-*α*, IL-1*β*, IL-6, and PGE2

TNF-*α* was quantified using a rat TNF-*α* kit (Invitrogen, USA), IL-1*β* was assessed using a rat IL-1*β* kit (R&D Systems, USA), IL-6 was evaluated using a rat IL-6 kit (Invitrogen, USA), and PGE2 was measured using an enzyme immunoassay kit (Biomatik, Canada). The optical density (OD) of all samples at 450 nm was measured using a Spectramax (M2, Molecular Devices, USA).

### 2.7. H&E Staining

The left knee joint was removed and fixed in Bouin solution for at least 24 h. Decalcification was conducted in a 2.5% nitric acid solution that was changed once a day for 7 days. The excised tissue was dehydrated using a tissue processor (Tissue- Tek® II, Japan), deparaffinized, stained with H&E (Muto, Japan), and observed under an optical microscope (Nikon, Japan).

### 2.8. Safranin O-Fast Stain

After deparaffinization, the right knee joint was reacted with Weigert's Iron Hematoxylin solution (Sigma, USA) for 10 min and stained with 0.001% fast green solution (Sigma, USA) for 5 min. The knee joint tissue was then reacted with 1% acetate solution for 10 s and stained with 0.1% safranin O solution (Sigma, USA) for 5 min. The tissue was then dehydrated and observed under an optical microscope (Nikon, Japan).

### 2.9. Dynamic Plantar Aesthesiometer

Rats were placed on a metal mesh table and adapted to the new environment for 5 min. The hind paw pressure withdrawal threshold was measured using a dynamic plantar aesthesiometer (37450, Ugo Basile, Italy) and expressed in grams. A mechanical stimulus was delivered to the plantar surface of the left hind paw below the floor of the plastic test chamber. A steel rod (0.5 mm diameter) was pushed against the hind paw with ascending force (from 0–50 g over 10 s). When the rat withdrew its hind paw, the mechanical stimulus stopped automatically and the force and time were recorded. Sensitivity of mechanical touch to the paws was measured on the day MIA was injected and after injection on treatment days 1, 3, 5, 7, 9, and 11.

### 2.10. Statistical Analysis

Data were analyzed using SAS 9.1 version for Windows by nonparametric Mann–Whitney *U* test. Statistical differences were evaluated using one-way ANOVA and results are expressed as means ± standard error (SE). Comparisons between groups were performed using the post hoc least significant differences (LSD) test. *P* < 0.05 and *P* < 0.01 were considered statistically significant.

## 3. Results

### 3.1. Quality Assessment of Three Marker Components in ChondroT

**Figure 2:**
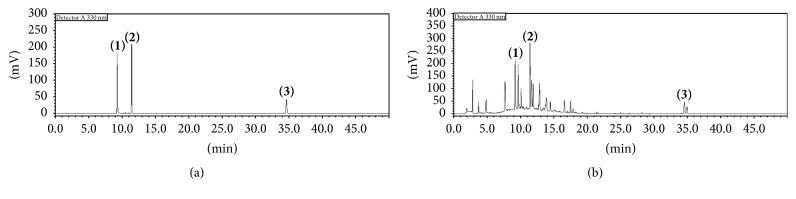
HPLC profile of a standard solution and ChondroT with detection at 330 nm. Chlorogenic acid (**1**), berberine Cl (**2**), and decursin (**3**).

The quality of ChondroT was assessed by HPLC using three marker compounds, including compound** 1** (Lonicerae Folium), compound** 2** (Phellodendri Cortex), and compound** 3** (Angelicae Gigantis Radix). All analytes were separated within 40 min, and a typical chromatogram of the 70% methanol extract of ChondroT is shown in Figure 2. Quantitation was achieved by UV-VIS detection at 330 nm based on retention time. The retention times of components** 1**–**3** were 9.25, 11.35, and 34.51 min, respectively. Under optimized chromatography conditions, the concentration of marker compounds** 1**–**3** in ChondroT was 3.67 ± 0.08, 2.41 ± 0.22, and 1.87 ± 0.18 mg/g, respectively (Table 2).

### 3.2. Effect of ChondroT on Paw and Knee Joint Swelling

**Figure 3:**
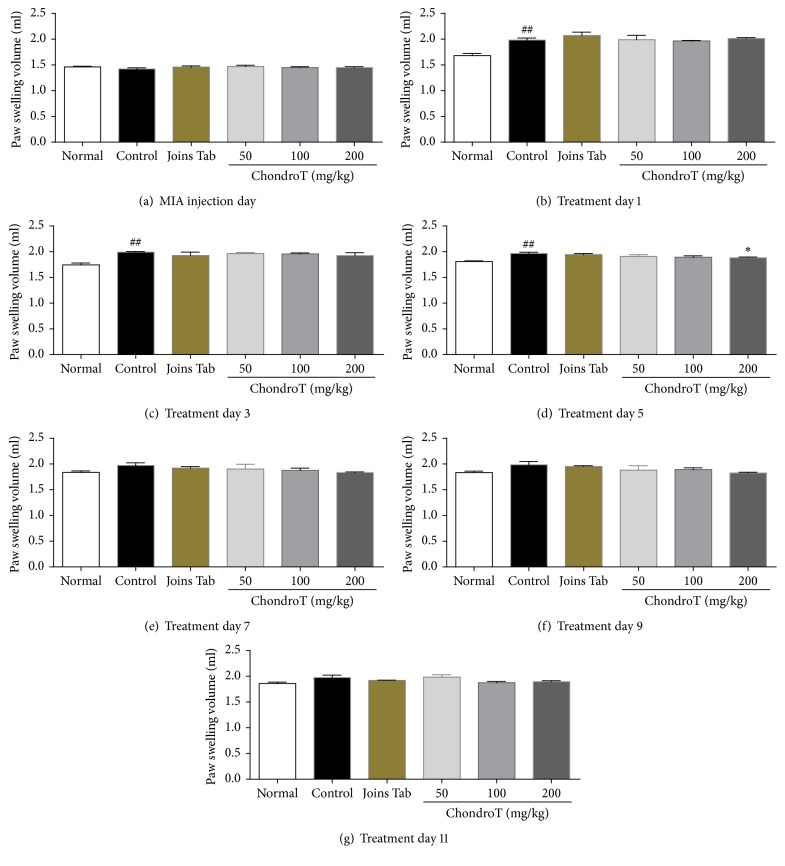
Effect of ChondroT treatment on paw swelling volume after ChondroT administration in monosodium iodoacetate- (MIA-) induced arthritic rats. MIA-induced arthritic rats were orally given water (control), Joins Tab (20 mg/kg), or ChondroT (50, 100, or 200 mg/kg). ^##^*P* < 0.01 compared with normal; ^*∗*^*P* < 0.05 compared with control.

Changes in paw and knee joint swelling are presented in Figure 3. Paw swelling was significantly greater on the 1st, 3rd, and 5th day of oral ChondroT administration in the control group than in the normal group. This volume was significantly less in the ChondroT 200 group on the 5th day than that of the control group (Figure 3).

### 3.3. Effect of ChondroT on Histopathological Changes

**Figure 4:**
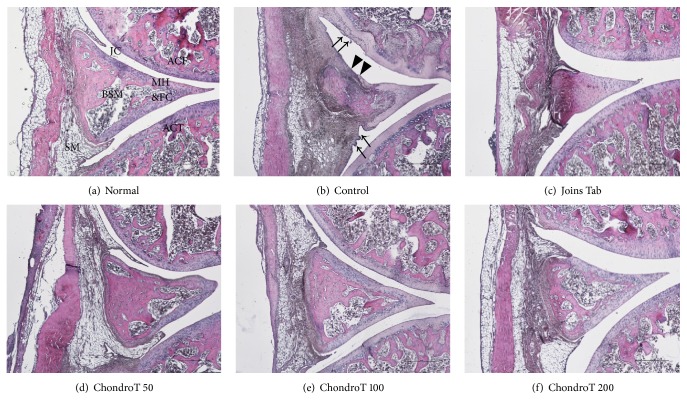
Histopathological changes (hematoxylin and eosin (H&E) staining) in the knee joint after ChondroT administration in monosodium iodoacetate- (MIA-) induced arthritic rats. Arrows (↓) indicate synovial membrane destruction. Arrow heads (▸) indicate compressed articular cartilage in the control. JC, joint cavity; ACF, articular cartilage of the femur; ACT, articular cartilage of the tibia; BSM, bony spicule within the meniscus; SM, synovial membrane; MH&FC, meniscus of hyaline and fibrocartilage. MIA-induced arthritic rats were orally given water (control), Joins Tab (20 mg/kg), or ChondroT (50, 100 or 200 mg/kg). Scale bars = 500 *μ*m.

Representative H&E stained histopathological lesions in the knee joint of normal; control; and ChondroT 50, 100, and 200 groups are shown in Figure 4. The control group showed partial damage of the synovial membrane, infiltration of inflammatory cells, and pannus formation (Figure 4(b)). Histopathological changes in the ChondroT 100 and 200 groups (Figures 4(e) and 4(f)) were improved over those observed in the control group. These ChondroT groups showed a uniform synovial membrane similar to that of the normal group and exhibited relatively little damage to the cartilage surface.

**Figure 5:**
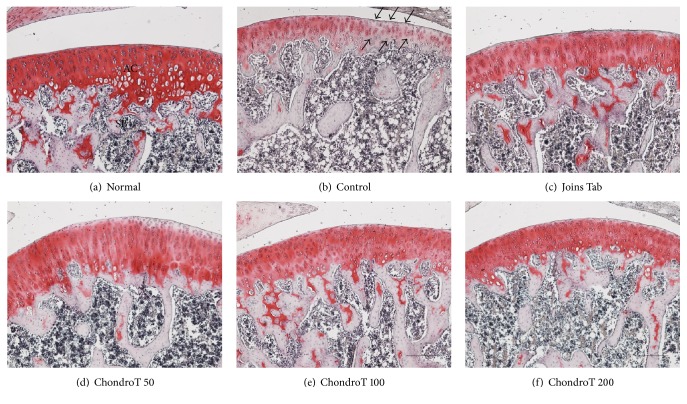
Histopathological changes (safranin O-fast staining) in the knee joint after ChondroT administration in monosodium iodoacetate- (MIA-) induced arthritic rats. Several shrunken nuclei (arrows ↓) were observed in the MIA control. JC, joint cavity; AC, articular cartilage; SB, spongy bone. MIA-induced arthritic rats were orally given water (control), Joins Tab (20 mg/kg), or ChondroT (50, 100, or 200 mg/kg). Scale bars = 100 *μ*m.

All ChondroT treated groups (50, 100, and 200 mg/kg), as well as the Joins Tab group, exhibited relatively higher levels of proteoglycans in the bone tissue and cartilage than that of the control group, which showed low proteoglycan levels, as determined by safranin O-fast staining (Figure 5).

### 3.4. Effect of ChondroT on the Plantar Withdrawal Response

**Figure 6:**
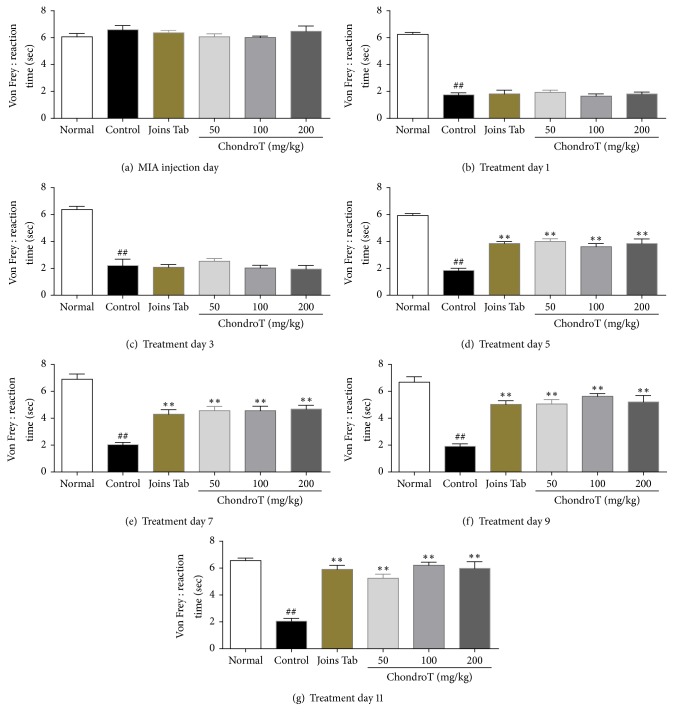
Effect of ChondroT treatment on the reaction time of plantar withdrawal response for all groups in monosodium iodoacetate- (MIA-) induced arthritic rats. The MIA-induced arthritic rats were orally given water (control), Joins Tab (20 mg/kg), or ChondroT (50, 100, or 200 mg/kg). ^##^*P* < 0.01 compared with normal. ^*∗∗*^*P* < 0.01 compared with control group.

Reaction time of the plantar withdrawal response was significantly higher in the ChondroT and Joins Tab groups than that of the control group from the 5th day to the 11th day after beginning treatment. In contrast, time to withdrawal was significantly lower in the control group than in the normal group from the 1st day to the 11th day after induction (Figure 6).

**Figure 7:**
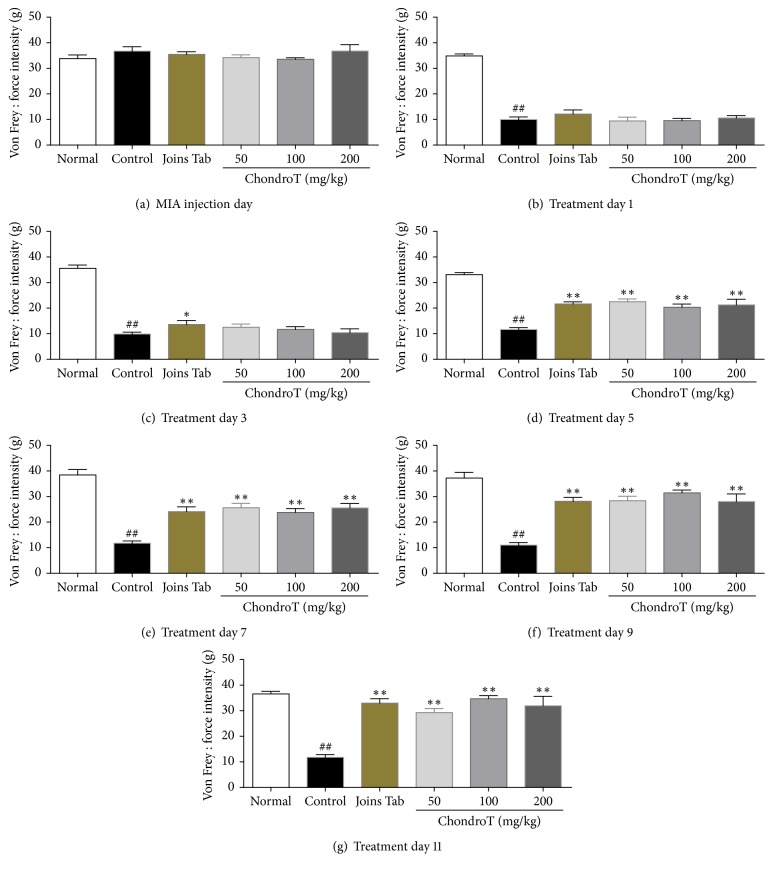
Effect of ChondroT treatment on the force intensity of plantar withdrawal response for all groups in monosodium iodoacetate- (MIA-) induced arthritic rats. MIA-induced arthritic rats were orally given water (control), Joins Tab (20 mg/kg), or ChondroT (50, 100, or 200 mg/kg). ^##^*P* < 0.01 compared with normal. ^*∗∗*^*P* < 0.01 and ^*∗*^*P* < 0.05 compared with control group.

The force intensity required to induce the plantar withdrawal response was significantly less in the control group than in the normal group at all measured time-points after treatment. The required force was significantly higher in the Joins Tab group from the 3rd to 11th day after treatment and in all ChondroT groups (50, 100, and 200 mg/kg) from the 5th to 11th day than that of the control group (Figure 7).

### 3.5. Effect of ChondroT on Proinflammatory Cytokines

**Figure 8:**
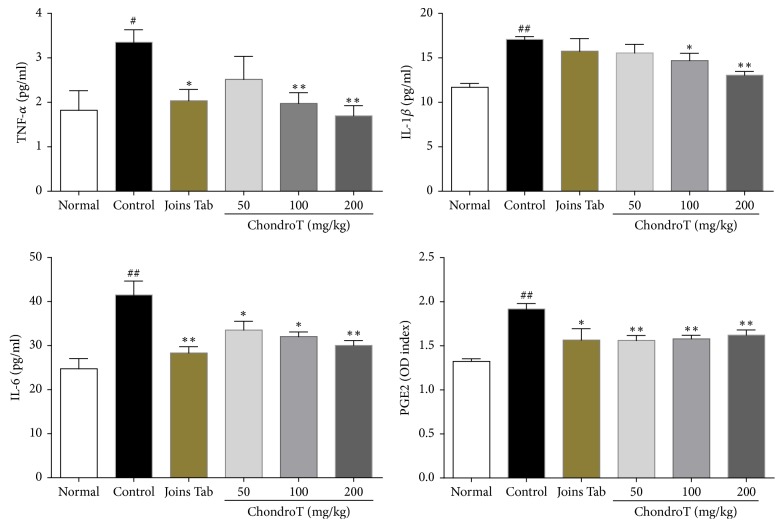
Effect of ChondroT treatment on tumor necrosis factor alpha (TNF-*α*), interleukin- (IL-) 1*β*, IL-6, and prostaglandin E2 (PGE2) concentrations after ChondroT administration in monosodium iodoacetate- (MIA-) induced arthritic rats. The MIA-induced arthritic rats were orally given water (control), Joins Tab (20 mg/kg), or ChondroT (50, 100, or 200 mg/kg). ^##^*P* < 0.01 and ^#^*P* < 0.05 compared with normal. ^*∗∗*^*P* < 0.01 and ^*∗*^*P* < 0.05 compared with the control group.

TNF-*α*, IL-6, IL-1*β*, and PGE2 levels in the control group were significantly higher than those of the normal group. TNF-*α* and IL-6 levels were significantly less in the ChondroT 100 and 200 groups and IL-1*β* and PGE2 levels were significantly less in all ChondroT treatment groups (50, 100, and 200 mg/kg) than those observed in the control group. TNF-*α*, IL-6, and PGE2 levels were also significantly lower in the Joins Tab group than in the control group (Figure 8).

### 3.6. Effect of ChondroT on Aminotransferase

**Figure 9:**
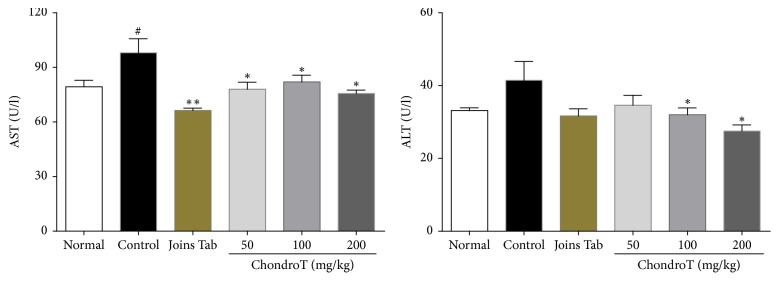
Effect of ChondroT treatment on serum aminotransferase concentrations after ChondroT administration in monosodium iodoacetate- (MIA-) induced arthritic rats. The MIA-induced arthritic rats were orally given water (control), Joins Tab (20 mg/kg), or ChondroT (50, 100, or 200 mg/kg). ^#^*P* < 0.05 compared with normal. ^*∗∗*^*P* < 0.01 and ^*∗*^*P* < 0.05 compared with control group.

Aminotransferase levels are indicated in Figure 9. ChondroT treated groups (50, 100, and 200 mg/kg) showed a significant decrease in AST. ALT levels were also significantly lower in the ChondroT 100 and 200 groups than in the control group. Further, AST and ALT levels were significantly lower in the Joins Tab group than in the control group (Figure 9).

### 3.7. Effect of ChondroT on Albumin, BUN, and Creatinine

There were no significant differences observed between rats in the ChondroT treatment groups (50, 100, and 200 mg/kg) and the control group.

## 4. Discussion

OA, a common and complex joint disease, is characterized by loss of articular cartilage, subchondral bone remodeling, bone spurs, ligament laxity, weakening of the periarticular muscles, and thickening of the capsule and synovial membrane. It is associated with pain, stiffness, and functional limitations [8]. Regarding pathogenesis, when a joint is loaded by weight, the cartilage cells, cartilage matrix, and subchondral bones become damaged by biochemical changes within the joint; disease occurs when the cartilage cells react to that damage, decreasing catabolism and increasing cartilage cell numbers [9].

A concrete method for halting OA progression has not yet been determined. The current goal is to improve joint function and quality of life while avoiding side effects and reducing pain through the prevention and correction of joint deformation [10]. Presently, treatments are divided into nonpharmacological conservative treatments, pharmacological treatments, and surgical treatments. In most cases, nonpharmacological conservative treatments are taken as a baseline and pharmacological treatment is added if necessary [11].

For this study, we used the MIA-induced OA model devised by Kalbhen [12]. In this model, pain, synovial proliferation, and loss of cartilage are induced by MIA, which decreases cellular glyceraldehyde-3-phosphate dehydrogenase activity when injected into the joint cavities of rats, chickens, rabbits, or horses. The model is often used in OA experiments because it allows for easy control of OA levels depending upon the injection concentration [13].

To objectively evaluate effects on the OA animal model using ChondroT in these experiments, a positive control group was established using Joins Tab, a natural medicine comprised of* Clematis mandshurica*,* Prunella vulgaris*, and* Trichosanthes kirilowii.* Joins Tab is used to treat OA, owing to its ability to suppress cartilage destruction and joint decomposition enzymes [14]. Although Joins Tab produces fewer side effects than existing synthetic medicines, it has still been reported to cause gastrointestinal disorders, kidney disorders, and adverse cardiovascular events in some individuals [15].

In these experiments, oral administration of ChondroT was initiated on the 8th day after arthritis induction and continued for 10 days thereafter. Podedema changes in the rats were observed in the control group and all drug treatment groups 1 day after drug administration. On the 5th day of drug treatment, a significant decrease in podedema was confirmed in the ChondroT 200 group. Podedema likely resulted from inflammation caused by MIA-induced OA [16]. These findings indicate that ChondroT administration aided recovery from OA.

When a mechanical stimulus is administered to cartilage, cartilage cells sense the damage or tissue changes and cytokines disrupt the balance between cartilage catabolism and anabolism. TNF-*α*, an upstream cytokine that is produced by macrophages and monocytes and participates in various cell activities, induces the secretion of various downstream inflammatory mediators, expands inflammation, restrains proteoglycan loss and resynthesis, and facilitates cartilage damage and OA progression. IL-1*β* is a potent cytokine that causes cartilage decomposition and facilitates cartilage matrix destruction, induces hyperalgesia, and stimulates the primary afferent nerve fibers, resulting in pain. IL-6, whose secretion is facilitated by IL-1 and TNF-*α*, is an acute regulatory protein and a factor involved in increased catabolism; it obstructs bone absorption and generation by facilitating osteoclast activity and mediating the joint inflammation reaction [9, 17, 18].

For these experiments, changes in TNF-*α*, IL-1*β*, and IL-6 were measured. The results show that administration of ChondroT in amounts greater than 100 mg/kg suppressed OA progression. Specifically, ChondroT significantly decreased all three factors (TNF-*α*, IL-1*β*, and IL-6), indicating that ChondroT is superior to Joins Tab, which only significantly decreased TNF-*α* and IL-6.

PGE2 is a major product created by the activity of inducible cyclooxygenase- (COX-) 2 that is known as a chief mediator of inflammation that induces vasodilation, pain, and fever; increases in PGE2 are known to parallel increases in tissue destruction observed during various inflammatory diseases, including OA [19]. A significant increase in PGE2 was observed in the control group, and significant decreases in PGE2 were observed in the ChondroT and Joins Tab groups, confirming the presence of an anti-inflammatory effect.

Regarding blood chemical changes attributable to drug administration, a significant decrease was confirmed for AST in all ChondroT groups, as well as the Joins Tab group. For ALT, a significant decrease was confirmed in the ChondroT 100 and 200 groups and the Joins Tab group. Regarding albumin, BUN, and creatinine, similar levels were observed in all experimental groups. There was no indication of liver or kidney damage caused by drug administration during these experiments, as no abnormal findings were found in the blood chemical tests.

Allodynia testing was conducted using a dynamic plantar aesthesiometer (the automated von Frey method). Reaction time (time from the moment a stimulator touches the sole of a subject until the subject breaks contact) and force intensity (the force at the moment the subject avoids the stimulator) were measured, and the results confirmed that the greatest significant increase over that of the control group occurred in the ChondroT 100 group on the 11th day after drug administration. Mechanical allodynia occurs in a MIA-induced OA model [20], and ChondroT attenuated this effect. In contrast, a significant decrease in reaction time and force intensity was consistently shown in the control group, indicating persistent allodynia and confirming that ChondroT administration effectively improves pain caused by OA.

Finally, the knee joints of rats were removed after the experiments to observe histopathological changes. H&E staining showed partial synovial damage, inflammatory cell infiltration, and pannus formation in the control group. However, in the ChondroT 100 and 200 groups, cartilage lacuna and cartilage cells together with uniform synovial observations similar to those in the normal healthy rat group were clearly present, with a relatively small degree of synovial joint damage. Safranin O-fast staining showed higher proteoglycan levels in the bone structure and adjacent inner cartilage layers in the ChondroT and Joins Tab groups than in the control group. Therefore, we confirmed that ChondroT administration is effective for inhibiting the progression of OA and for protecting joints.

## 5. Conclusions

In summary, the greatest effects were observed in the MIA-induced OA animals treated with ChondroT 200. To further confirm the effectiveness of ChondroT 200, systematic clinical studies should be conducted through supplemental experiments and consistent studies. If its effectiveness is further proven, this Korean medicine will contribute to OA treatment in the future.

## Figures and Tables

**Table 1:** Composition of ChondroT and the used parts of 5 herbs.

Latin name	Scientific name	Family	Used part	Rate	Source
Osterici Radix	*Ostericum koreanum* Maximowicz	Umbelliferae	Root	6	Korea
Angelicae Gigantis Radix	*Angelica gigas* Nakai	Umbelliferae	Root	4	Korea
Clematidis Radix	*Clematis mandshurica* Ruprecht	Ranunculaceae	Root	4	China
Lonicerae Folium	*Lonicera japonica* Thunberg	Caprifoliaceae	Leaf	4	China
Phellodendri Cortex	*Phellodendron amurense* Ruprecht	Rutaceae	Tree bark	3	China

**Table 2:** Concentration of the three marker components in ChondroT as determined by HPLC (*n* = 3).

Compound	Mean (mg/g)	SD	RSD (%)	Source
Chlorogenic acid	3.67	0.08	2.20	*L. japonica*
Berberine Cl	2.41	0.22	8.99	*P. amurense*
Decursin	1.87	0.18	9.57	*A. gigas*
